# Thermoelectric Properties of Nano-Meso-Micro β-MnO_2_ Powders as a Function of Electrical Resistance

**DOI:** 10.1186/s11671-015-1000-6

**Published:** 2015-07-15

**Authors:** Morgan Hedden, Nick Francis, Jason T Haraldsen, Towfiq Ahmed, Costel Constantin

**Affiliations:** Department Physics and Astronomy, James Madison University, 901 Carrier Drive, 22807 Harrisonburg, Virginia USA; Los Alamos National Laboratory, Theoretical Division, 87545 Los Alamos, New Mexico

**Keywords:** Thermoelectric properties, Transition metal oxides, Manganese oxide, Seebeck coefficient, Figure of merit, Particle size versus electrical resistance, 84.60Rb, 72.15.Cz, 81.05.Hd

## Abstract

Particle sizes of manganese oxide (β-MnO_2_) powders were modified by using a mortar and pestle ground method for period of times that varied between 15–60 min. Particle size versus ground time clearly shows the existence of a size-induced regime transition (i.e., regime I and II). Thermoelectric properties of β-MnO_2_ powders as a function of electrical resistance in the range of *R*_*P*_ = 10 - 80*Ω* were measured. Based on the data presented, we propose a model for the β-MnO_2_ system in which nanometer-scale MnO_2_ crystallites bond together through weak van der Waals forces to form larger conglomerates that span in size from nanometer to micrometer scale.

## Background

The environmental impact resulting from the use of fossil fuel as an energy source affects the entire globe. Eventually, fossil fuels will no longer be a reasonable source of energy, and alternative energy sources will be needed. Thermoelectric materials (TE) that directly convert heat into electricity are a viable option to replace the conventional fossil fuel because they are reliable, cost effective, and use no moving parts. The efficiency of a TE material is defined by the dimensionless figure of merit, ZT = (*σ* ⋅ *S*^2^ ⋅ *T*)/(*k*_*E*_ + *k*_*L*_), where *S* is the Seebeck coefficient, σ is the electrical conductivity, *T* is the temperature, and *k*_*E*_ and *k*_*L*_ are the carrier and lattice thermal conductivities, respectively. One of the most known and commercially available TE material is bismuth telluride (Bi_2_Te_3_), which exhibits one of the highest ZT values at room temperature (i.e., ZT ~1). Thermoelectric devices (TED) made out of Bi_2_Te_3_ are already commercially available and are used for small-scale energy harvesting [1]. However, one of the main drawbacks of Bi_2_Te_3_ is the fact that it is poisonous. Transition metal oxides (TMO) are attractive materials for replacing Bi_2_Te_3_ because they are non-toxic, inexpensive, withstand high temperature, and have minimal impact on the environment [2]. Walia et al. have written a recent, and excellent, review about the thermoelectric properties of TMOs [3].

In particular, manganese dioxide (MnO_2_)-based materials are of great interest for various applications, ranging from catalysts and batteries to energy-efficient devices and carbon-storage applications [4–7]. Mn atoms are multivalent, and thus form oxides with several different stoichiometries [8]. At room temperature and atmospheric pressure, the most stable phase is β-MnO_2_, which crystalizes in a pyrolusite (or a rutile) crystal structure, but other metastable phases such as α and γ exist as well [3, 9–13]. Song et al. have demonstrated a TE generator that lit up a regular light-emitting diode by using β-MnO_2_ powders [12]. He found a giant *S* coefficient in the range of 20–40 mV/K as a function of particle size and electrical resistance (i.e., 30 − 120 *KΩ*). This discovery ignited interest in using β-MnO_2_ even as the core TE material in a thermopower wave source demonstrated by Ref. [14]. Although the semiconducting properties of MnO_2_ are well known (Ref. [11]), not too much is known about the TE properties of this system. In the literature, there are six research groups that reported electrical conductivities and *S* coefficient values for MnO_2_ powders (i.e., Refs. [9–14]). However, out of these works, only the Ref. [14] reported thermal conductivity values.

In this work, we present TE properties of β-MnO_2_ powders as a function of electrical resistance. The particle size of β-MnO_2_ powders were modified by a pestle and mortar for periods of time that varied between 15–60 min. A size-induced phase change was observed between particles that were ground between 15 and 30 min. We correlated the measured TE properties with the particle electrical resistance in the range of *R*_p_ = 10 ÷ 80*Ω*. The largest *S* coefficient, largest power factor, and smallest thermal conductivity values were found to be *S* = 316 *μV*/*K*, and *σ* ⋅ *S*^2^ = 5.8 × 10^− 7^W/(*m* ⋅ *K*^2^), all observed at particle electrical resistance of. *R*_*p*_ = 9.8*Ω* From these values, we calculated the highest figure of merit to be ZT = 3.28 × 10^− 4^.

## Methods

### Particle Size Modification

**Fig. 1:**
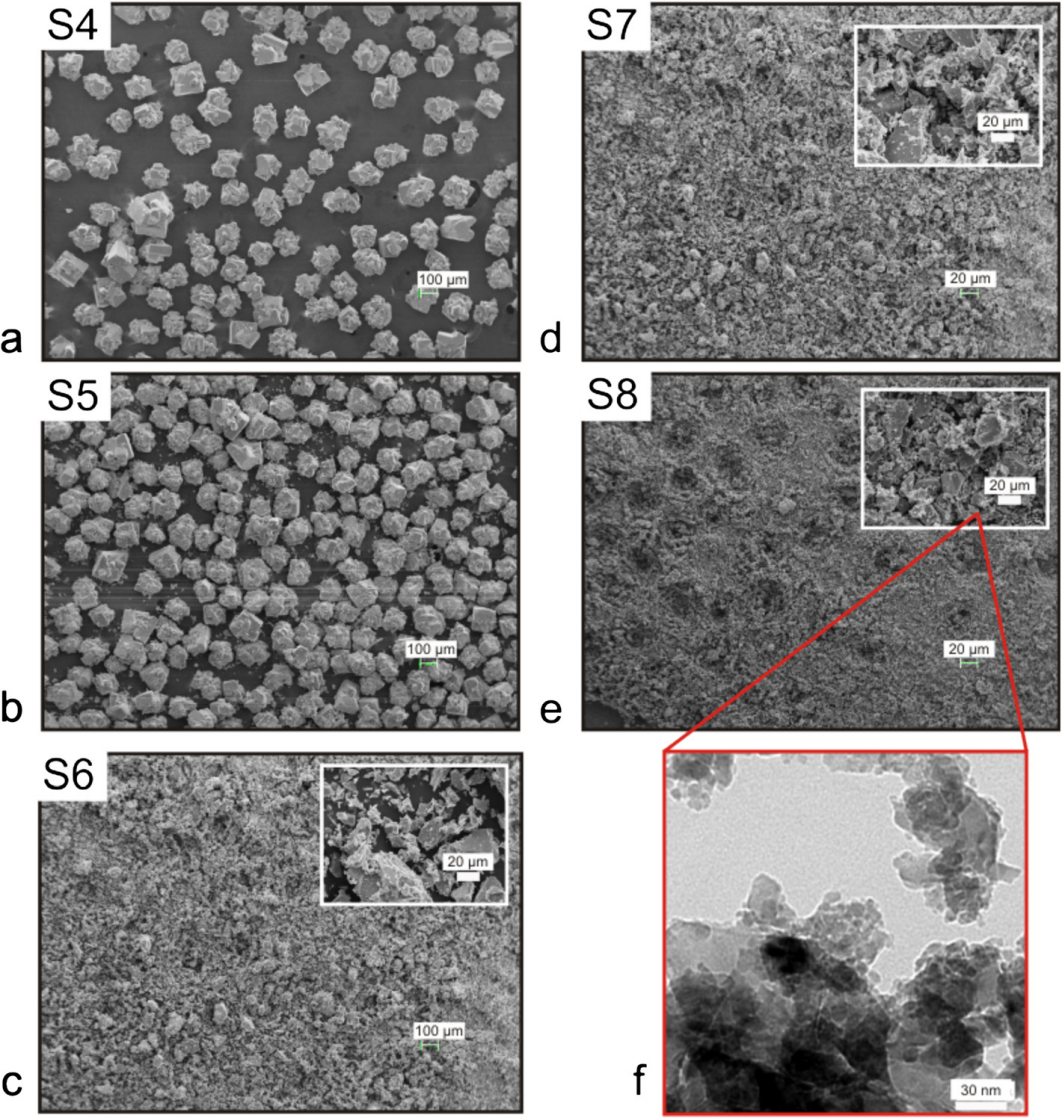
Images **a–e** are experimental SEM results of MnO_2_ particles for samples S4, S5, S6, S7 and S8. Image **f** shows experimental TEM of particles for sample S8

**Fig. 2:**
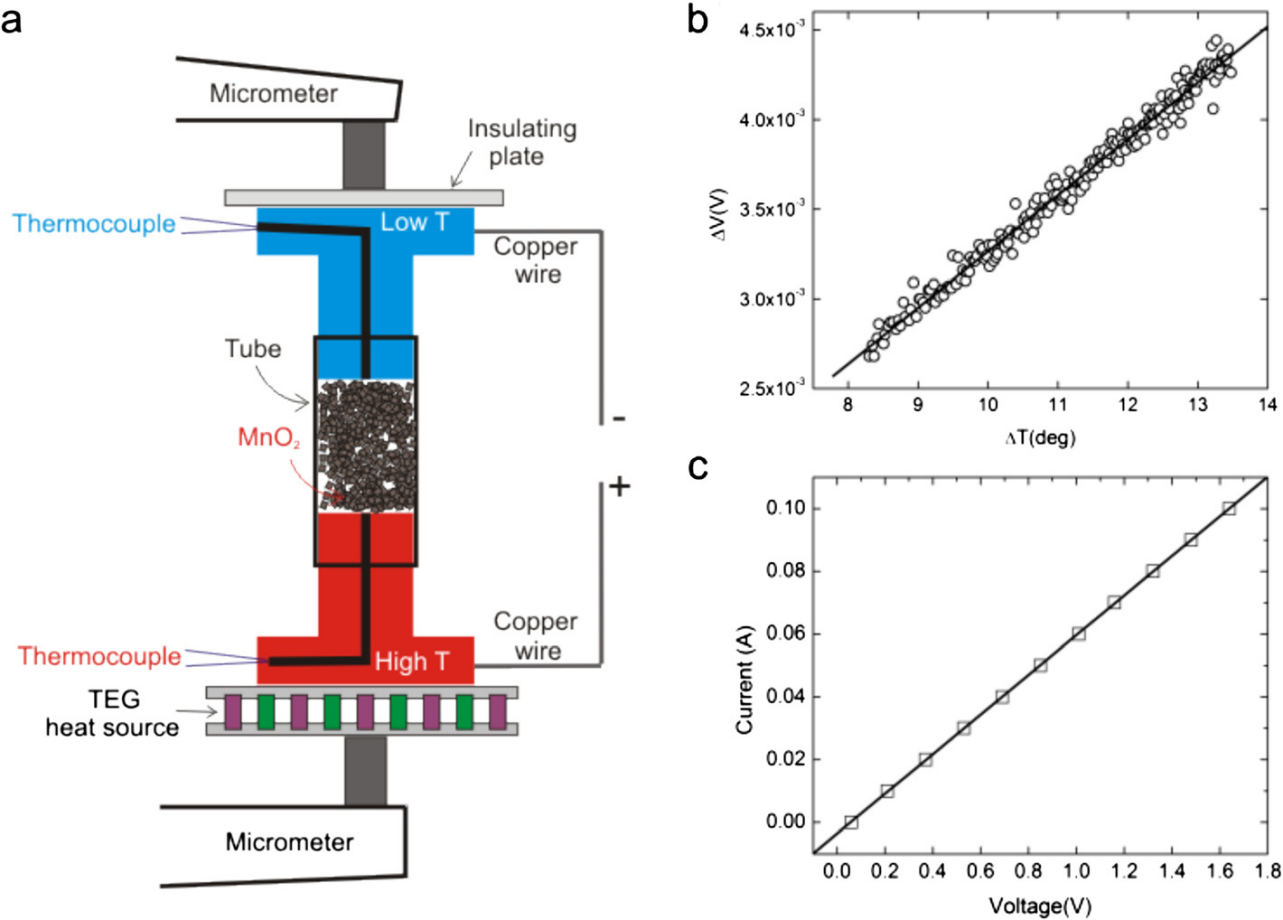
Experimental setup used for measurements of Seebeck coefficient (*S*) and electrical conductivity (*σ*) (**a**). A typical plot of *S* measurement performed with a homemade LabVIEW software (**b**). A typical plot of an IV measurement that was taken manually (**c**)

In this work, we purchased commercially available MnO_2_ powders (from Sigma Aldrich, 60–230 mesh), and we ground five samples by a mortar and pestle method to have varying particle sizes. We kept the sample S4 in its original size (i.e., not modified) (Fig. 1a). The rest of the samples (i.e., S5 through S8) were ground for increased increments of 15 min each (Fig. 2b–e). The altered samples were hand ground in a ceramic-coated mortar and pestle containing 95 % ethyl alcohol, 190 proof (95 %) with spectrophotometric grade (commercially available from Fisher Scientific, catalog number AC61511-0010).

### Apparatus

Fig. 2a shows the apparatus used to measure Seebeck coefficient and electrical conductivity. The MnO_2_ particles were placed in a plastic tube between two copper plugs. The tube has an inner diameter of 14.34 mm, and the copper plugs were lathed down to size to ensure that the particles would not escape from the tube. The small diameter of the plugs is 20 mm while the larger diameter is 35 mm and the height is 5 mm. A commercially available, high-temperature thermoelectric generator (TEG) device was placed on the bottom plug while an insulating plate was placed on the top plug. A hole large enough to fit a thermocouple was drilled down through the center of both plugs until approximately 1 mm of copper was separating the end of the hole and the particles (Fig. 2a). A K-type thermocouple was placed in each of these holes to monitor temperature, and the whole setup was then placed between a micrometer. By adjusting the height read of the micrometer, we induced a pressure onto the samples. This pressure applied was used to vary the particle density and *R*_P_. All the samples were measured at RT and atmospheric pressure and at an electrical resistance range of *R*_P_ = 10–80Ω. A similar setup for measuring the *S* coefficient was also used by Ref. [12].

### Seebeck Measurements

The Seebeck coefficient (*S*) is a measure of the magnitude of an induced thermoelectric voltage in response to a temperature difference across the material (i.e., *S* = − *ΔV*/*ΔT*). We measured *S* by using the setup shown in Fig. 1a. For creating a temperature difference between the bottom copper plug and the top copper plug, a TEG was plugged into a DC voltage source and set at 2.334 V (Fig. 2a). The thermocouples, along with soldered copper wires, were connected to a data collection unit interfaced to LabVIEW to allow measurement of voltage and temperature difference between the copper plugs (Fig. 2a). The source was then turned on for 90 s. After 90 s, measurements of voltage and temperature difference were taken until the temperature difference between the copper plugs was *ΔT* ≥ 5 *K* (Fig. 2a). The thermocouples monitored the temperature gradient across the particles, and the copper wires soldered to the plugs read the *S* voltage produced. The data collected in LabVIEW was then plotted to determine the *S* coefficient, and a typical measurement for sample S4 is shown in Fig. 2b (i.e., *S* = − 313*μV*/*K* at a resistance of *R*_p_ = 73.3*Ω*).

### Electrical Conductivity Measurements

Electrical conductivity (σ) was calculated by using *σ* = *x*/(*R*_p_ ⋅ *A*), where *x* is the measured tube distance occupied by MnO_2_ particles, *R*_P_ is the measured MnO_2_ particle resistance extracted from I/V plots, and *A* is the cross-section area occupied by MnO_2_ particles which coincides with the cross-section area of the plastic tube (Fig. 2a). A typical I/V curve is presented in Fig. 1c for sample S4, and the calculated resistance was *R*_p_ = 17*Ω*.

### Thermal Conductivity Measurements

Thermal conductivity (*k*) measurements were performed by ThermTest Inc. (Ref. [15]), and samples S4 and S6 were measured at two different densities each (Table 1). ThermTest used, for our measurements, the TPS 2200 Thermal Constants Analyzer instrument, which uses the transient plane source method explained elsewhere [16].

**Table 1:** Thermal conductivities of samples S4 and S6 at different packing densities

Sample	Thermal conductivity	Density(kg/m3)
S4	0.2096±0.0013	2503
S4	0.5153±0.005	2700
S6	0.3417±0.0008	3100
S6	0.5137±0.0008	3500

## Results and Discussion

Figure 1 shows scanning electron microscopy (SEM) images of samples S4, S5, S6, S7, and S8. Transmission electron microscopy (TEM) of sample S8 is shown in Fig. 1f. Other works modified (or obtained) the MnO_2_ particles by different methods, including ball milling (Ref. [12]), hydrolytically deposited powder (Ref. [11]), pyrolytic techniques (Ref. [9, 13]), and reduction processes (Ref. [10]). We chose to modify our MnO2 particles by a simple mortar and pestle method as explained in part A of the Methods section. ImageJ [17] software was used to measure all average dimensions assuming that the particles had cubic symmetry in 3D (or square symmetry in 2D) (Table 2). The as-received average particle size of sample S4 was *d*_41_ = 140 ± 2*μm*. After 15 min of the particle size modification procedure, sample S5 showed three different sizes, namely *d*_51_ = 112 ± 10*μm*, *d*_52_ = 136 ± 7*μm*, and *d*_53_ = 12.0 ± 0.1*μm*. It is clear that there was only a ~ 2.9 % decrease from the initial size of 140*μm* to 136*μm* in sample S5 and, small flakes which broke off of the larger particles started to appear (Fig. 2b). Sample S6 showed two particle sizes, namely *d*_61_ = 24.5 ± 0.8*μm*, and *d*_62_ = 12 ± 0.1*μm*. We noticed that, for this set of samples, between samples S5 and S6, we recorded the largest particle decrease change of ~ 82 % from 136*μm* to 24.5*μm*. The decrease in the small particle sizes from *d*_53_ to *d*_62_ was only ~ 2.4 % (Fig. 2c). For sample S7, we found two particle sizes (i.e., *d*_71_ = 23 ± 1*μm* and *d*_72_ = 1.06 ± 0.02*μm*). In this case, from sample S6 to S7, the small particle sizes decreased the most (i.e., from 12.0*μm* to 1.06*μm*, a 91.5 % decrease) (Fig. 1d). Lastly, for sample S8, there were two particle sizes measured at *μm* scale (i.e., *d*_81_ = 5.14 ± 0.09*μm* and *d*_82_ = 0.83 ± 0.03*μm*) and a third particle size at nm scale (i.e., *d*_83_ = 6 ± 1*μm*). The TEM image from Fig. 1f shows a closer view of sample S8. It is clear to see that the smaller particles form large conglomerates and the larger particles tend to bunch with the even bigger conglomerates.

**Table 2:** Particle sizes of samples S4, S5, S6, S7, and S8 as measured for Fig. 2a–e

	^a^	^b^			^c^		^d^		^e^		^f^
Sample	S4	S5			S6		S7		S8		
size	1	1	2	3	1	2	1	2	1	2	3
Particle size (μm)	140±2	112±10	136±7	12.3±0.4	24.5±0.8	12.0±0.1	23±1	1.06±0.02	5.14±0.09	0.83±0.03	0.006±0.001

In order to calculate the particle sizes, we assumed a cubic symmetry (3D), or in our case, a square (2D) symmetry for the MnO_2_ particles because our particles exhibit a rutile β-MnO_2_ crystal structure. The particle sizes tabulated here were given as a side length of a square, and it was calculated as the square root of the particle areas

**Fig. 3:**
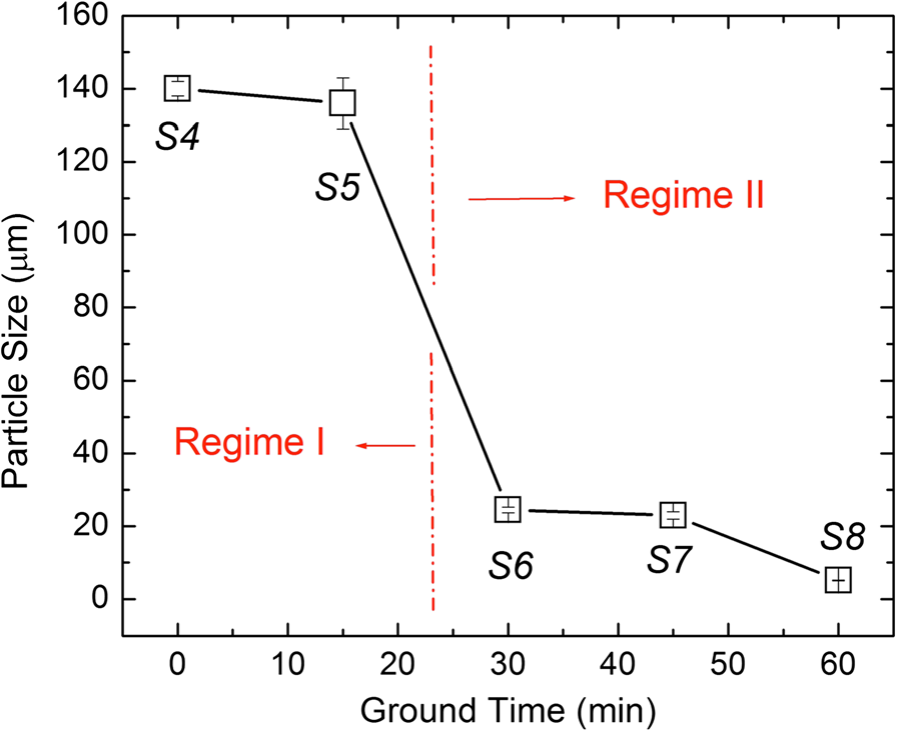
Plot of particle sizes (μm) versus ground time (min) of samples S4, S5, S6, S7, and S8. This data is also presented in Table 2; however, in this plot, we present the changes in the largest particle sizes so we can emphasize the existence of two particle size regimes. Explicitly, particle sizes are very close for sample S4 and S5 (i.e., regime I), and similar behavior can be seen for samples S6, S7, and S8 (i.e., regime II)

In order to paint a better picture for to the particle-size modification procedure, we plotted in Fig. 3 the particle size (μm) versus ground time (min). This data is also presented in Table 2, with the exception that we chose to plot only the larger particle sizes for each sample. For example, we plotted size 1 for samples S4, S6, S7, and S8, and size 2 for sample S5 [Table 2]. It is interesting to note that based on the change in particle size, we discovered two different size regimes (i.e., regime I, and regime II shown in Fig. 3). These size-induced regimes agree very well with our data of particle electrical resistance versus tube length (Fig. 5a) and Seebeck versus particle electrical resistance (Fig. 5b). These two distinct regimes are also obvious by comparing the particle morphology between samples S5 and S6. For example, sample S5 still retains a lot of large particles at the 100-μm scale inherited from sample S4 whereas samples S6, S7, and S8 have no particles at this scale. Based on the SEM/TEM images presented in Fig. 1, we presume that within each MnO_2_ conglomerate, we can distinguish a large variety of particle sizes starting from nanometer scale all the way to hundreds on micrometers.

**Fig. 4:**
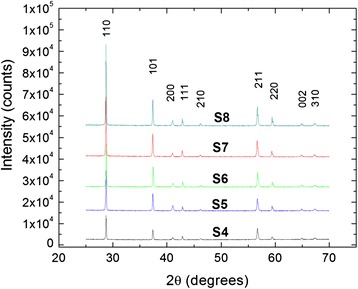
Experimental X-ray diffraction results of samples S4, S5, S6, S7, and S8. Sample S4 was the as-received sample, while samples S5, S6, S7, and S8 were prepared by hand grinding with a mortar and pestle for 15, 30, 45, and 60 min., respectively

Shown in Fig. 4 are the main peaks seen in XRD over the range 25°< 2θ° < 70° for MnO_2_ powders for samples S4, S5, S6, S7, and S8, respectively. The XRD was performed with a PANalytical X’Pert PRO MPD theta-theta diffractometer with X-ray radiation of Cu (wavelength of *λ* = 1.54060 Å for *Cu K*_α1_ peak). The samples were back-mounted on a 16*-*mm diameter standard powder-sample holder and rotated with an angular speed of 3 RPM with a scan time step of 100 s. The peaks observed at 2θ ~ 28.7°, 37.4°, 41.0°, 42.8°, 46.1°, 56.7°, 59.4°, 64.9°, and 67.3° correspond to the crystallographic directions of [110], [101], [200], [111], [210], [211], [220], [002], and [310], respectively. The position and orientation of these peaks confirmed that our particles have β-MnO_2_ crystal structure (i.e., pyrolusite) (Ref. 12). This crystal structure is part of the rutile tetragonal group, and it is composed of parallel chains of octahedrons made up of manganese ions that are each surrounded by six O_2_ atoms. It is well known from the literature that MnO_2_ has three distinct phases (α, β, and γ) with different properties, and from a TE standpoint, β-MnO_2_ is preferred since the electrical conductivity of α-phase MnO_2_ is approximately six orders of magnitude lower [18]. Although in Fig. 3 where we observe the existence of two regimes (i.e., regime I and II) of particle size as a function of ground time, there is no correlated crystalline phase change. The pyrolusite crystal structure stays constant for all samples, which implies that the ground process only alters the particle size and not the crystalline phase.

Extracting information about the crystalline size based on the broadening of the XRD peaks is not a trivial task. It involves understanding diffraction peak broadening and also choosing the right method of analysis [19]. For example, the broadening of diffraction peaks occurs due to five factors. The first factor is due to instrumental effects that include non-ideal optics, wavelength dispersion, sample transparency, axial divergence, flat sample effect, and detector resolution. The second factor is due to the crystallite size broadening which varies inversely with crystallite size. It is worth noticing that a crystallite size is (most often!) not the same with particle size. The third factor involves the existence of microstrain within each crystal, which can be due to multiple reasons including non-uniform lattice distortions, stacking faults, lattice dislocations, antiphase domain boundaries, and grain-surface relaxations. The fourth factor has to do with solid solution inhomogeneity, and the fifth factor is related to temperature variation. Another point for consideration is the analysis of XRD peak broadening effects, which involve different methods that, sometimes, yield conflicting results. The three main methods used for analysis are the following: 1) simplified integral-breadth, 2) Fourier, and 3) double-Voigt. Most researchers use the simplified integral-breadth method, which assumes either a Gaussian of Lorentzian function for a size- and/or strain-broadened profile, but it is widely accepted today that the double-Voigt approach, that is a Voigt-function approximation for both size-broadened and strain-broadened profile, is a better model than the simplified integral-breadth method [19]. Although the double-Voigt method is the most superior of all the analysis methods mentioned above, a combination of a simplified integral-breadth method together with independent microscopy measurements such as SEM and/or TEM are widely accepted. A good example of such a combination is given by the work of A. K. Zak et al. who prepared zinc oxide nanoparticles by a sol-gel combustion method and found that the estimated nanoparticle size from the TEM, Williamson-Hall, and the size-strain method were in very good agreement [20]. Regarding the MnO_2_ system, the group of Wallia et al. [14] claimed that they obtained MnO_2_ nanopowder by using ball-milling method with crystals ranging from 400 to 700 nm in size; however, their SEM micrograph shows particle conglomerates that are ranging in size from a few hundreds of nm up to 2 μm. They present no XRD results that would support their crystalline size claim. The groups of Xia et al. [10] and Preisler et al. [11] obtained γ-MnO_2_ powders, but no supporting SEM/TEM or XRD measurements are presented. Finally, the group of Song et al. [12] reports particle sizes of 100–500 nm with thicknesses of around 30 nm. Although, they present XRD and SEM measurements; a close inspection of their SEM concludes that the average particles sizes exceeds 500 nm. Also, they used only the low-angle peak of (110) orientation to calculate the particle size through Scherrer equation, and they claimed a particle size of 30 nm in diameter. A close inspection of their XRD data show that the full width at half maximum (FWHM) of the second order peak of (110) (i.e., the peak (211) shown in Fig. 2 of their work) has a lower value of FWHM as compared to the (110) peak, which clearly indicates the nonhomogenity nature of their powders.

Just to solidify our findings, we also used Scherrer formula to estimate the particle size. The Scherrer formula is *D* = (*k* ⋅ *λ*)/(*β* ⋅ cos(*θ*)), where *D* is the particle size in nanometers, *λ* is the wavelength of X-ray radiation (i.e., 1.54060 Å for *Cu K*α1 radiation), *k* is a constant equal to 0.94, and *β* is the measured FWHM of the peak, and *θ* is the peak position shown in Fig. 4. Based on our data, obtaining information on only the (110) XRD peak does not hold reliable results. For example, the particle size for sample S4 (i.e., the as-received powder) showed *D*_4_ = 306.30 nm smaller than the particle size for sample S6 (i.e., the sample that was ground for 30 min) *D*_6_ = 546.26 nm. Therefore, it is important that the data should be obtained from more than one peak in order to get consistent and reliable results.

**Fig. 5:**
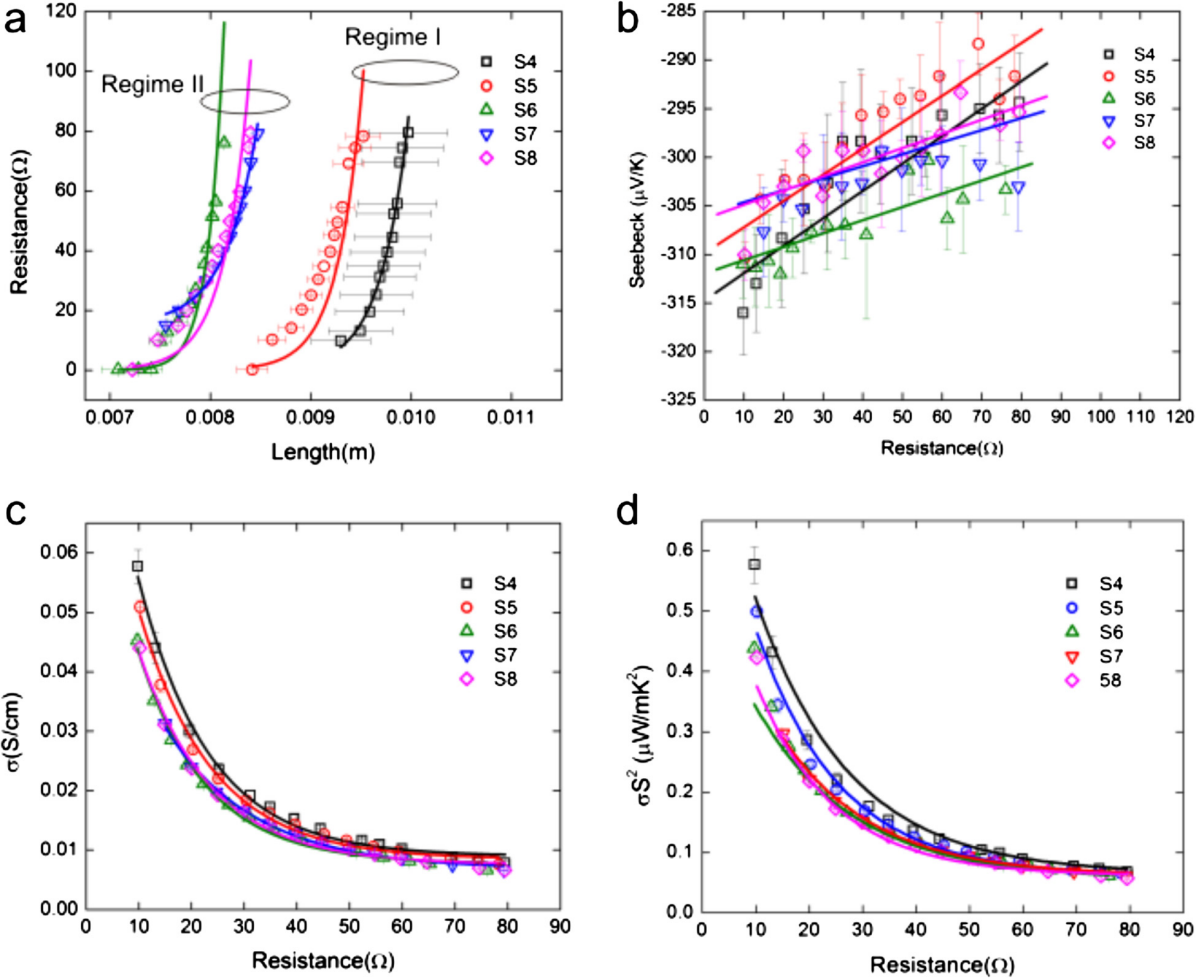
Experimental electrical resistance versus MnO_2_ powder length (**a**), *S* versus resistance (**b**), σ versus resistance (**c**), and power factor (PF) versus resistance (**d**). The electrical resistance here is denoted in the text as *R*_P_

Figure 5a shows that the electrical resistance (*R*_P_), for samples S4 through S8, varies exponentially with tube length (*L*). We believe that this response induces further exponential behavior observed in σ and power factor (*σ* ⋅ *S*^2^) versus *R*_P_ (Fig. 5c, and d). Tube length also tends to saturate for sample S6 at a value of ∼ 0.012 *m*, and again, here, we observe the existence of regime I and regime II, which agree with our finding in Fig. 3.

In Figure 5b we present the Seebeck coefficient (*S*) versus electrical resistance. The *S* measured varies linearly with *R*_P_, and it has the tendency to have larger values at smaller resistances (Fig. 5b). Seebeck coefficient is also observed to separate into the two regimes as mentioned before, because samples S4 and S5 have steeper slopes as compared to samples S6, S7, and S8 (Fig. 5b). To date, there is no report in the literature that performed such a systematic study of thermoelectric properties versus electrical resistance for the MnO_2_ system.

The largest (and smallest) *S* was measured for samples S4 (S5) at values of − 316*μV*/*K* (−288*μV*/*K*) and at resistances of 9.8 *Ω* (69*Ω*) (Fig. 5b). Our absolute values for *S* agree very well with reported values by works of Refs. [10, 11, 13, 14] (i.e., |*S*| = 71 - 273*μV*/*K*) (Table 3). However, we were not able to see the giant *S* observed by Song and co-workers [12]. This is understandable if we consider the range of resistance values that we measured at (i.e., *R*_p_ = 10 - 80 *Ω*) as compared to Song’s resistance values (i.e., *R* = 30 - 120 *KΩ*). Also, our method of particle modification was quite different. For example, we used a simple mortar and pestle (i.e., explained in the particle modification section) while Song et al. used a ball-milling method. Furthermore, our data also suggests that the *S* increases with decreasing *R*_P_, which is opposite to the work of Song et al. (i.e., *S* = − 20, 000*μV*/*V* measured at *R* = 30 *KΩ*) and (i.e. *S* = − 40, 000 *μV*/*K* measured at *R* = 120 *KΩ*) (Table 3) (Ref. [12]).

**Table 3:** MnO_2_ crystalline phase, conductivity (σ), Seebeck coefficient (*S*), power factor (*σ* S^2^), thermal conductivity (*k*), figure of merit (*Z*), unitless figure of merit (ZT) and temperature (*T*) for this work and literature

MnO_2_ phase	*σ*(*S*/*cm*)	*S*(*μV*/*K*)	*σS* ^*2*^(*W*/*m*∙*K* ^*2*^)	*κ*(*W*/*m*∙*K*)	*Z*(1/*K*)	*ZT*	*T*(*K*)	References
β (NP)	1.00 × 10	−1.90 × 10^3^	3.61 × 10^−3^	4.0	9.0 × 10^−4^	5.6 × 10^−1^	623	[14]
β (TF)	1.00 × 10	−3.00 × 10^2^	9.00 × 10^−5^	–	–	–	300	[9]
γ (P)	8.20 × 10^−1^	−3.06 × 10^2^	7.68 × 10^−6^	–	–	–	300	[10]
γ (P)	1.79	−2.00×10^2^	7.16 × 10^−6^	–	–	–	300	[11]
β (NP)	7.64×10^−5^	−3.00×10^4^	6.88 × 10^−6^	–	–	–	300	[12]
β (NP)	1.27 × 10^−4^	−2.00 × 10^4^	5.09 × 10^−6^	–	–	–	300	[12]
β (NP)	3.18 × 10^−5^	−4.00 × 10^4^	5.09 × 10^−6^	–	–	–	300	[12]
S4 β (NMμ)	5.77 × 10^−2^	−3.16 × 10^2^	5.76 × 10^−7^	0.5153	1.12 × 10^−6^	3.28 × 10^−4^	293	This work
S6 β (NMμ)	4.53 × 10^−2^	−3.11 × 10^2^	4.38 × 10^−7^	0.5137	8.53 × 10^−7^	2.50 × 10^−4^	293	This work
S4 β (NMμ)	7.79 × 10^−3^	−2.94 × 10^2^	6.75 × 10^−8^	0.2096	3.22 × 10^−7^	9.46 × 10^−5^	294	This work
S6 β (NMμ)	6.64 × 10^−3^	−3.03 × 10^2^	6.10 × 10^−8^	0.3417	1.78 × 10^−7^	5.23 × 10^−5^	293	This work
Mn(OH)_2_ (TF)	2.00 × 10^−6^	−1.29 × 10	3.33 × 10^−14^	–	–	–	523	[13]
Mn(OH)_2_ (TF)	2.09 × 10^−6^	−1.23 × 10	3.16 × 10^−14^	–	–	–	473	[13]
γ (TF)	2.24 × 10^−6^	−7.0	1.1 × 10^−14^	–	–	–	573	[13]

*NP* stands for nanoparticle, *P* stands for powder, *TF* stands for thin film, NMμ stands for nano-meso-micro

Figure 5c shows σ versus *R*_P_ for samples S4 through S8. We find that the largest (and smallest) σ data were measured for samples S4 (S8) at values of (0.058 *S*/cm) and at resistances of *R*_p_ = 9.8 *Ω* (*R*_p_ = 79 *Ω*).

In Figure 5(d), we present the power factors (*σ* ⋅ *S*^2^) versus *R*_P_. The largest (and smallest) power factors we had obtained came for samples S4 (S8) 5.8 × 10^− 7^ *W*/(*m* ⋅ *K*^2^) 5.7 × 10^− 8^ *W*/(*m* ⋅ *K*^2^) at resistances of *R*_p_ = 9.8 *Ω* ⋅ (*R*_*p*_ = 79*Ω*).

**Fig. 6:**
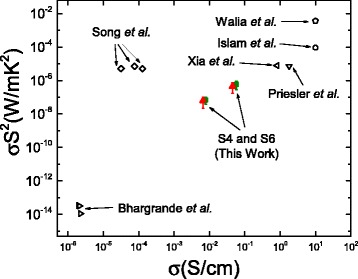
Literature (experimental) data for PFs versus σ (also this data is presented in Table 3). Our results from samples S4 and S6 are presented in *solid green* and *red triangles*. Both axes are plotted in log scales

In Fig. 6**,** we present the literature data compared to our data for power factor versus electrical conductivity. Our power factor values are lower than other works by Walia et al. (i.e., 3.61 × 10^− 3^ *W*/(*m* ⋅ *K*^2^)) (Ref. [14]), Islam et al. (i.e., 9 × 10^− 5^ *W*/(*m* ⋅ *K*^2^)) (Ref. [9]), Xia et al. (i.e., 7.68 × 10^− 6^ *W*/(*m* ⋅ *K*^2^)) (Ref. [10]), Priesler et al. (i.e., 7.16 × 10^− 6^ *W*/(*m* ⋅ *K*^2^)) (Ref. [11]), and Song et al. (i.e., 6.88 × 10^− 6^ *W*/(*m* ⋅ *K*^2^) and 5.09 × 10^− 6^ *W*/(*m* ⋅ *K*^2^)) (Ref. [12]). It is worth noticing that only works of Walia et al. and ours have reported values for *k* so far. Although the Walia et al. particle modification procedure was similar to the works of Song et al., they did not observe the giant *S* as observed by the work of Song et al. (Ref. [12]).

In Table 3, we present values for figure of merit *Z* (*1*/*K*) and the unitless figure of merit ZT that were reported in the literature. It appears that the highest *Z* and ZT values were obtained by the work of Walia et al. (i.e., *Z* = 9 × 10^− 4^1/*K* and ZT = 5.6 × 10^− 1^). If we were to assume a thermal conductivity value of *k* = 0.2096 (i.e., our lowest *k* values measured) for the other works that did not report thermal conductivity (Table 3). Still the highest *Z* and ZT values would belong to the work of Walia et al. [14] followed by the works of Islam et al. (i.e., *Z* = 4.29 × 10^− 4^1/*K* and ZT = 1.29 × 10^− 1^) [9], Xia et al. (i.e., *Z* = 3.66 × 10^− 5^1/*K* and ZT = 1.1 × 10^− 2^) [10], Priesler et al. (i.e., *Z* = 3.42 × 10^− 5^1/*K* and ZT = 1.02 × 10^− 2^) [11], Song et al. (i.e., *Z* = (7.29 - 9.84) × 10^− 3^1/*K* and ZT = (2.43 - 3.28) × 10^− 5^) [12], and Bhargrande et al. (i.e., *Z* = (0.254 - 1.59) × 10^− 13^1/*K* and ZT = (3.0 - 8.3) × 10^− 11^) [13]. A closer look at the literature available has shown that works who have reported high conductivities also have had the highest power factor values (Refs. [14, 9, 10]). While Song et al. [12] reported giant *S* values of |*S*| = 20,000 - 40,000 *μV*/*V*, their reported electrical conductivity was *σ* = (3.18 - 12.7) × 10^− 5^ *S*/*cm*, which has a strong contribution toward lowering their ZT values.

**Fig. 7:**
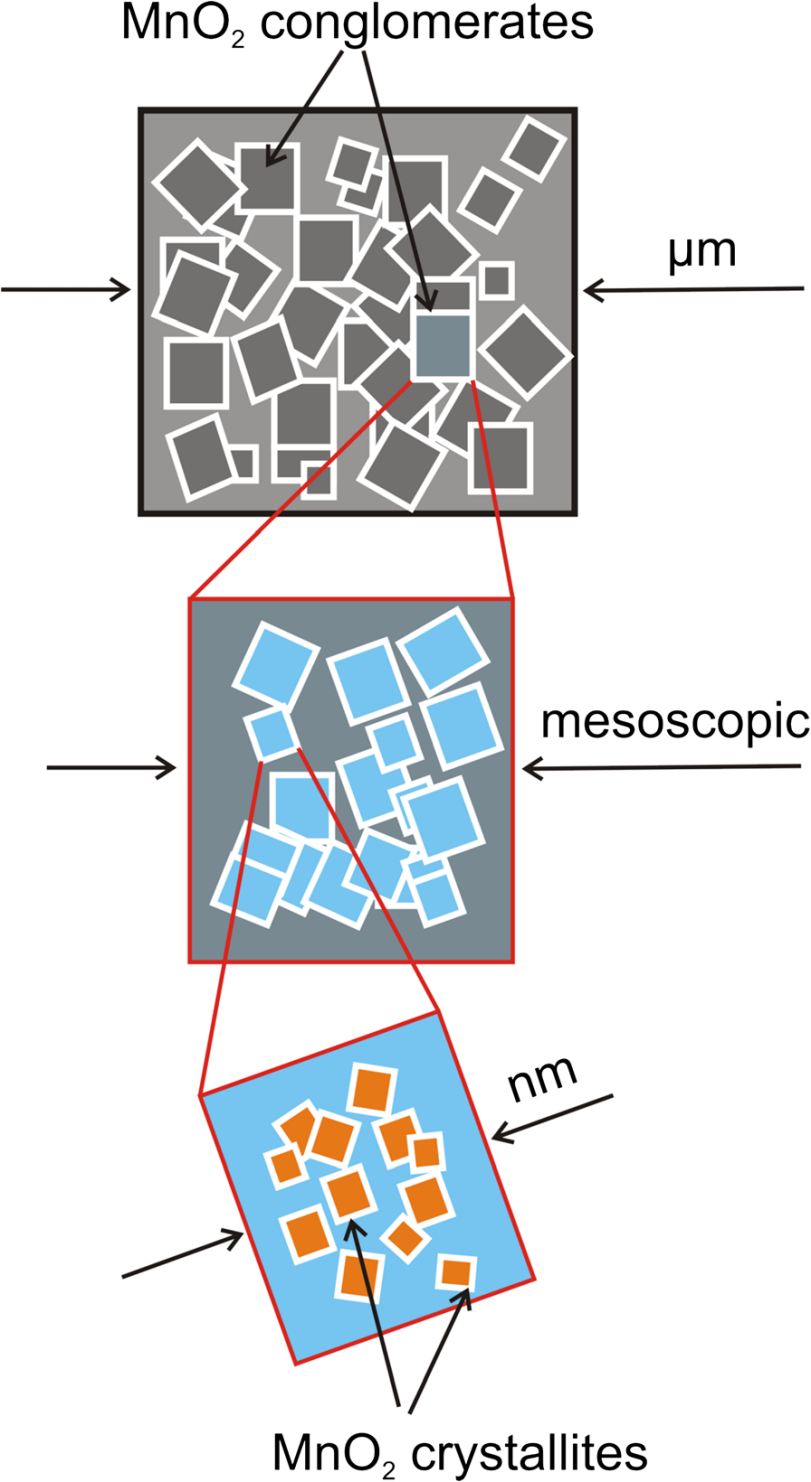
Proposed model for the MnO_2_ conglomerates

All our data lead us to believe that our β−MnO_2_ system behaves differently as compared to other works. For example, the work of Song et al. [12] might have reported particle sizes at nanometer scale with a high degree of homogeneity, whereas in our case, we have observed particle sizes ranging from nanometer all the way to hundreds of micrometers, all coexisting within the same sample. That is why we propose the model shown in Fig. 7, in which smaller MnO_2_ crystallites at nanometer scale bond together through weak van der waals forces to form larger conglomerates that span anywhere from mesoscopic to microscopic scale.

## Conclusions

We have investigated thermoelectric properties of β-MnO_2_ powders as a function of electrical resistance in the range of *R* = 10 - 80*Ω*. We found two distinct particle size regimes (i.e., regime I and II), which were further confirmed by our thermoelectric measurements. According to SEM and TEM data, most of the MnO_2_ show a wide range of crystallite sizes that span from nanometer all the way to micrometer sizes. The data presented suggest that the thermoelectric properties of β-MnO_2_ depend heavily on particle size distribution and particle morphology. The details of particle agglomeration is presented as a model in which smaller MnO_2_ crystallites (at nanometer scale) bond together through weak van der Waals forces to form larger conglomerates with sizes ranging from mesoscopic to microscopic scale. Future research consists in a systematic study of thermoelectric properties as a function of high-energy ball-milling process parameters such as ground time, and angular speed.

## Abbreviations

TE: thermoelectric material
Z: figure of merit
ZT: unitless figure of merit
TED: thermoelectric devices
TMO: transition metal oxides
MnO_2_: manganese oxide
SEM: scanning electron microscopy
TEM: transmission electron microscopy
TEG: thermoelectric generator
S: Seebeck coefficient
σ: electrical conductivity
XRD: X-ray diffraction
PRO: professional
MPD: materials-powder diffractometer
